# Gait and cognition in older adults: Insights from the Bronx and Kerala

**DOI:** 10.4103/0972-2327.74253

**Published:** 2010-12

**Authors:** Anne F. Ambrose, Mohan L. Noone, V. G. Pradeep, Beena Johnson, K. A. Salam, Joe Verghese

**Affiliations:** Department of Rehabilitation Medicine, Mt Sinai Medical Center, New York, NY, USA; 1 Department of Neurological Sciences, Baby Memorial Hospital, Calicut, Kerala, India; 2 Department of Neurology, Albert Einstein College of Medicine, Bronx, NY, USA

**Keywords:** Dementia, elderly, gait, speed

## Abstract

**Background:**

Recent reports indicate that gait dysfunction can occur early in the course of cognitive decline suggesting that motor and cognitive functions in older adults may share common underlying brain substrates, pathological processes, and risk factors.

**Objective:**

This study was designed to report the association between gait and cognition in older adults in USA and the southern Indian state of Kerala.

**Materials and Methods:**

Literature review of gait and cognition studies conducted in Bronx County, USA as well as preliminary results from the Kerala-Einstein study (Kozhikode city, Kerala).

**Results:**

Review of published studies based in the Bronx shows that both clinical and quantitative gait dysfunction are common in older adults with cognitive impairment. Furthermore, clinical and quantitative gait dysfunction in cognitively normal older adults was a strong predictor of future cognitive decline and dementia. Our preliminary study in Kozhikode city shows that timed gait is slower in older adults diagnosed with dementia and mild cognitive impairment syndrome compared to healthy older controls.

**Conclusions:**

A strong association between gait and cognition is seen in seniors in USA as well as Kerala. A better understanding of the relationship between gait and cognition may help improve current diagnostic and therapeutic approaches globally.

## Introduction

The ability to walk is an index of health and functional status in older adults.[1,2] At age 60, 85% of community residing seniors have normal gait; however, this proportion drops to 18% by age 85.[3] Walking (gait) is important for maintaining autonomy and independence. While reliant on the functioning of peripheral organs such as the musculoskeletal system, vestibular apparatus, and cardiovascular fitness, normal gait performance also depends on cortical input.[4,5] The degree of cortical involvement in gait control varies from minimal input required during steady-state walking to increased input provided in the presence of environmental perturbations such as uneven pavements or while walking and talking.[4–6]

Efficient gait patterns require generation of coordinated lower-extremity movements, thought to be generated by a central pattern generator that is then modified by higher cortical and reflexive spinal programs under the control of several gait centers in the brainstem, cerebellum, and cortex.[4] Hence, it is not too surprising that gait and cognition are both impaired together in many diseases associated with aging such as the various dementia syndromes or Parkinson’s disease that affect parts of the nervous system that control both motor and cognitive functions.[7–9] There is growing interest in defining abnormalities of gait as an early clinical marker for cognitive decline and dementia in older adults.[7–9] Gait performance in older adults has been operationalized in many ways including self-report,[10] slow gait,[11,12] inability to walk a fixed distance,[11,12] distance covered in a fixed time,[13] or using clinical gait classifications.[3,8,9] To better understand the relationship between gait and cognition function in aging and dementia, we conducted a literature review of aging studies conducted in Bronx County, USA as well as discussed preliminary results from the Kerala-Einstein study (Kozhikode city, Kerala).

## Clinical Assessment of Gait

Despite its widespread use in clinical practice,[1–3,8,9] there are no universally accepted gait classifications. In our studies based in the Bronx,[3,9] we use a clinical gait classification to categorize gait abnormalities. This gait classification is a part of the standard neurological examination that also tests cranial nerves, strength, sensation, and deep tendon reflexes.[3,9] Gait is clinically rated as either “normal” or “abnormal” following visual inspection of walking patterns. Abnormal gaits are further classified as either neurological or nonneurological (due to causes such as arthritis or dyspnea). Neurological gaits are subtyped as unsteady if two or more of the following features are present: marked swaying or losing balance while walking in a straight line, in tandem, or making turns. Ataxic (cerebellar) gait is wide-based and unsteady with other cerebellar signs such as intention tremor or inability to perform rapid alternating movements. Patients with neuropathic gaits have unilateral or bilateral foot drop with associated signs such as sensory loss and depressed or absent deep tendon reflexes. Frontal gait is characterized by short steps, wide base, and difficulty in lifting the feet off the floor. Older adults with Parkinsonian gaits have small shuffling steps, flexed posture, absent arm swing, en bloc turns, and festination. Patients with hemiparetic gait swing their leg outward and in a semicircle from the hip (“circumduction”). In spastic gait both legs circumduct, and when severe cross in front of one another (“scissoring”). Video weblinks of the abnormal gait subtypes are available.[9] This clinical gait classification has established test–retest reliability and predictive validity.[3,9] Unlike our gait classification, the reliability and validity of most other descriptive gait classifications have not been verified and subtypes overlap.[7,8] For instance, marche a petit pas, cautious gait, and apractic gait are used to describe gait abnormalities that share clinical features such as slowness, short steps, or wide base with frontal gaits.[3,7,8,14]

Prevalence of abnormal gait in our Bronx cohort using this classification was 35.0% (95% CI: 28.6–42.1).[3] Incidence data of gait disorders, which are crucial for assessing the association of sociodemographic and medical risk factors with abnormal gait, have been lacking. Incidence of abnormal gait was 168.6 per 1000 person-years (95% CI: 117.4–242.0) in our Bronx cohort, and increased with age.[3] While there was no sex differences in the incidence of abnormal gaits overall, men had a higher incidence of neurological gaits and women had a higher incidence of nonneurological gaits. This sex difference may relate to differing medical risk factors. For instance, arthritis is more common in older women and is a major contributor to nonneurological gait abnormalities.[3] We plan to determine if similar sex differences in gait subtypes are seen in Kerala seniors in our ongoing Kerala-Einstein study (see below).

## Quantitative Assessment of Gait

While an integral aspect of patient evaluation, clinical gait assessments have several limitations. Most assessment protocols are not standardized or validated. Most gait abnormalities are mild and detection is dependent on the examiner’s expertise.[3] Quantitative gait assessments, independent of clinical diagnosis, may help avoid these shortcomings.[15] Often in the past, the technical details of gait analysis made clinical gait analysis extremely cumbersome and time-consuming. Recent advances, such as improved computer processing and the development of passive as opposed to active marker systems, have enabled the faster acquisition of kinematic data without heavy encumbering attachments and wires trailing from the subject.[15]

In our studies, we use a computerized walkway with embedded pressure sensors (GAITRite, CIR Systems, Havertown, PA, USA) at study visits.[15] Subjects are asked to walk on the mat at their “normal pace” for two trials in a quiet well-lit hallway wearing comfortable footwear and without any attached monitors. On the basis of footfalls recorded on the walkway, the software automatically computes gait variables as the mean of two trials. We select eight gait parameters to report based on their associations with adverse health outcomes such as dementia and falls reported in our and other studies;[11,14–16] velocity (cm/s), cadence (steps/min), stride length (cm), swing time (s), stance time (s), and double support phase (%). Standard deviation (SD) of stride length and swing time was used as proxies for gait variability.[15–17] Table 1 provides definitions of these commonly used gait variables.

**Table 1:** Definition of quantitative gait parameters

Variable	Unit	Definition
Velocity	cm/s	Distance covered on two trials by the ambulation time
Stride length	cm	Distance between heel points of two consecutive footfalls of the same foot. Variability in length between strides is reported as standard deviation.
Cadence	Steps/min	Number of steps taken in a minute
Double support	s	Time elapsed between first contact of current footfall and the last contact of previous footfall, added to the time elapsed between the last contact of current footfall and the first contact of next footfall
Swing time	s	Duration when the foot in the air and is the time taken from toe off to heel strike of the same foot. Variability in swing time is reported as standard deviation
Stance time	s	Duration when the foot is on the ground and is the time taken from heel strike to toe off of the same foot

All quantitative parameters described below are automatically calculated as the mean of two trials by the gait software (adapted from ref. 8).

We have subjected these eight selected gait variables to factor analysis to derive three factors that represent gait domains of pace, rhythm, and variability.[15–17] This additional approach accounts for the collinearity between these gait variables, which would prevent the assessment of their relative contributions to various outcomes in the same statistical model. This approach also allows us to explore specific gait domains.[15–17]

**Figure 1:**
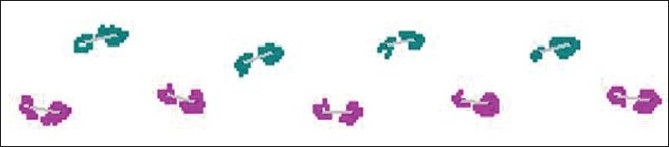
Footfall patterns recorded on the instrumented walkway of an older adult patient with frontal gait. The wide base can be readily visualized.

Figure 1 provides an example of footfalls of a patient with frontal gait; the wide base can be readily visualized. The GAITRite system is widely used in clinical and research settings, and excellent reliability has been reported in our and other centers.[6,15–17]

## Gait Dysfunction and Cognitive Impairment Syndromes

### Mild cognitive impairment

Mild cognitive impairment (MCI) is a diagnosis given to individuals who have cognitive impairments beyond that expected for their age and education, but that do not interfere significantly with their daily activities.[18] Cognitive impairments may be in either memory (amnestic) or nonmemory (nonamnestic) domains.[18] MCI is considered to be a transitional stage between normal aging and dementia. Worse fine and complex motor skills, equilibrium, and limb coordination have been reported in older adults with MCI.[19,20] On the other hand, another study reported no differences in multiple motor measures in subjects with MCI compared to controls.[21] However, this study was modest in sample size and included only patients presenting to a memory clinic with memory complaints.[21]

Quantitative testing revealed gait dysfunction in subjects with both amnestic and nonamnestic MCI subtypes compared to healthy controls in our Bronx cohort.[17] Neurological gaits were more almost twice more common in amnestic MCI than in normal controls.[17] Subjects with MCI and gait abnormalities (defined as either having slow gait velocity or neurological gait) were more disabled than subjects with MCI without gait abnormalities.[17]

### Dementia

We examined the relationship between neurological gaits and risk of dementia in 422 nondemented participants between ages 75 and 85 years in the Bronx Aging Study; 85 (20%) with neurological gait abnormalities at baseline.[9] Over 21 years follow-up, 125 subjects developed dementia, 70 Alzheimer’s disease, and 47 vascular dementia. Thirty-seven of the 85 (43%) subjects with neurological gaits and 88 of the 337 (26%) subjects with normal gait went on to develop dementia over subsequent follow-up. The hazard ratio of developing any type of dementia in patients with neurological gait abnormalities was 2.03 (95% CI: 1.39–2.99), and the hazard ratio of developing non-Alzheimer’s dementia was 3.75 (95% CI: 2.20–6.38). Abnormal gait was not a significant predictor of Alzheimer’s disease. Among the neurologic gait subtypes, unsteady, frontal, and hemiparetic gaits were associated with an increased risk of developing non-Alzheimer’s dementia. Investigators from the Sydney Older Persons Study also reported that elderly patients with a combination of cognitive, vascular, and extrapyramidal features including abnormal gait were at increased risk of progression to dementia over a 3-year follow-up period.[22]

To develop a clinically useful approach to predict vascular dementia over shorter intervals, we defined a “high-risk neurological gait” syndrome based on the presence of any one of hemiparetic, frontal, and unsteady gaits (described above).[23] At baseline, 54 subjects out of the 399 eligible nondemented older adults in the Bronx Aging Study had “high-risk neurological gait” syndrome using this criteria. Fourteen subjects developed vascular dementia over 3 years and 25 by the end of 5-year follow-up. High-risk neurological gaits predicted risk of vascular dementia within the first 3 years (hazard ratio compared to remaining subjects 3.3, 95% CI: 1.8–5.9) and 5 years (hazard ratio 2.7, 95% CI: 1.7–4.2) of follow-up. Adding information on executive dysfunction (based on the Digit Symbol Substitution test scores at the baseline visit) to the syndrome improved its predictive validity for vascular dementia. Hence, diagnosing “high-risk neurological gait” syndrome provides a clinically relevant approach to identifying older adults at high risk of developing vascular dementia over relatively short follow-up intervals.

### Quantitative gait

We reported the association of quantitative gait parameters in the Einstein Aging, a community-based cohort study in the Bronx.[15] Factor analysis was used to reduce eight baseline quantitative gait parameters to three independent factors representing pace, rhythm, and variability.[15] The pace factor had strong loadings on velocity and stride length, the rhythm factor on cadence and timing, and variability factor loaded heavily on gait “variability” measures. These factors could be considered to represent separate and independent gait domains.

A one-point increase on both the rhythm (by 48%) and variability factors (by 37%) predicted risk of developing dementia.[15] The pace factor predicted decline in executive function, the cognitive domain primarily involved in vascular dementia.[15] A one-point increase on the pace factor predicted decline on executive function measured by the digit symbol substitution (by 29%) and letter fluency tests (by 92%).[15] Gait rhythm predicted memory decline (by 107%),[15] which can start many years before Alzheimer’s disease is diagnosed.

Alzheimer involvement of the motor cortex is said to occur only late in the disease process and gait disturbances early in the disease is considered an exclusion criterion. Parkinsonian gait in the absence of idiopathic Parkinson’s disease has been correlated with substantia nigra neurofibrillary tangles even in cases without clinical Alzheimer’s disease or with minimal Alzheimer pathology.[24] These findings raise the possibility that Alzheimer pathology may involve brain regions regulating gait early, but the subtle quantitative gait abnormalities may be overshadowed by behavioral symptoms in the early clinical stages of Alzheimer’s disease.

It is assumed that stride length and velocity (pace) are controlled supraspinally by phasic output from the basal ganglia to the supplementary motor area, whereas spinal and brainstem mechanisms may determine cadence (rhythm).[4] Neural substrates of gait variability are less understood.[25] It has been suggested that regulation of gait variability is automated and requires minimal cognitive input in healthy adults, but may be perturbed in the presence of disease.[25]

## Kerala-Einstein Study Experience

The goal of the Kerala-Einstein study funded by the National Institutes of Health, USA, is to identify risk factors for cognitive decline in Kerala seniors. We examined gait characteristics in 217 adults aged 60 and older enrolled from the Neurology clinics at the Baby Memorial Hospital, Kozhikode, over a 15-month period from 2008 to 2010. Eligible subjects received detailed evaluation of general cognitive status as well as specific cognitive domains such as memory, attention, executive function, and language using standard neuropsychological tests. Clinical and neuropsychological data were reviewed at consensus case conferences by study clinicians and diagnosis assigned using established criteria. Following consensus diagnostic procedures, 52 subjects were diagnosed with dementia (33 Alzheimer’s disease, 13 vascular dementia, 5 mixed dementia, and 1 insufficient information to subtype), 19 with MCI (13 amnestic subtype and 3 nonamnestic subtype), and 146 were normal controls.

Subjects with dementia were older (mean age, 71.2 years) than those with MCI (mean age, 69.3 years) and cognitively normal controls (mean age, 66.9 years). There were also a higher prevalence of men in all three groups in this clinic-based sample; dementia (60.3%), MCI (63.2%), and controls (60.2%).

**Figure 2:**
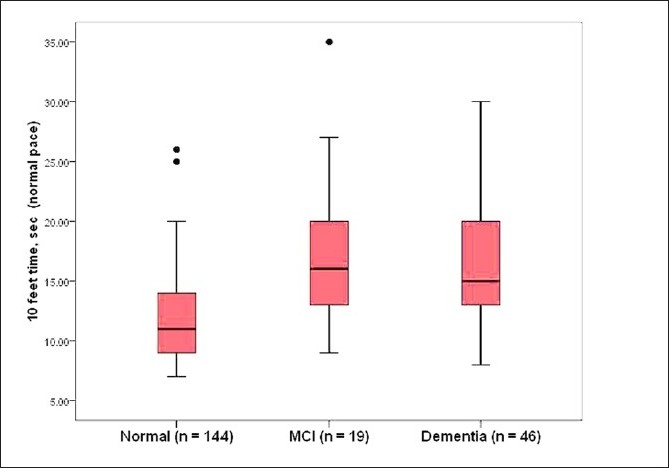
Boxplots depicting walking time over the 10-foot course at usual pace in subjects with dementia, MCI, and normal controls in the Kerala-Einstein study. Higher times indicate worse performance. The line in the middle of the box represents the median value. The ends of the box represent the 25th and 75th quartile values. The bars show the range of scores and black dots are outliers.

**Figure 3:**
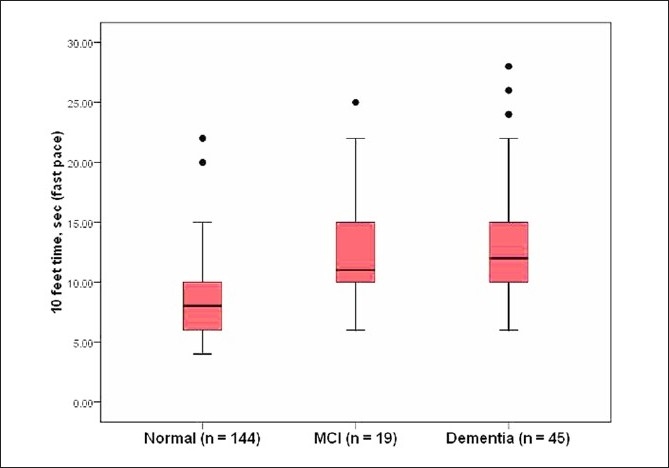
Boxplots depicting walking time over the 10-foot course at fast pace in subjects with dementia, MCI, and normal controls in the Kerala-Einstein study.

Subjects were timed with a stopwatch while walking a 10-foot course at their usual pace as well as at a fast pace. Six out of the 52 subjects with dementia and two out of the normal controls were unable to walk, and were not included in this analysis. An additional subject with dementia walked the course at usual pace, but not at fast pace. The remaining 46 subjects with dementia (median 15 s, *P* < 0.001) and 19 MCI subjects (median, 16 s, *P* < 0.001) walked slower at their usual pace over the 10-foot course compared to the 144 normal controls (median, 11 s). A similar pattern was seen comparing the 45 ambulatory subjects with dementia (median, 12 s, *P* < 0.001) and the 19 MCI subjects (median 11 s, *P* < 0.001) to the 144 controls (median, 8 s) when they walked at a fast pace over the same distance. Figures 2 and 3 present the time to walk 10 feet (longer times worse) at usual pace as well as at fast pace by diagnostic groups.

These findings replicate our experience in our Bronx cohort described earlier. The presence of slow gait in older subjects is helpful in identifying not only physical disability, but also cognitive impairments, and should prompt further investigations to identify underlying causes.

## Clinical Perspectives

Our longitudinal studies in the Bronx and preliminary studies in Kozhikode describe strong associations between gait and cognition at cross-section as well as longitudinally. Despite being considered an integral part of the standard clinical evaluation, gait assessments are often neglected in the evaluation and management process when evaluating patients with suspected cognitive impairment or dementia. Our studies show that simple clinical observations of walking patterns can help identify older adults at higher risk for cognitive decline in clinical settings. Although our quantitative studies in the Bronx were conducted using a computerized walkway, gait velocity can also be simply assessed (as in our Kozhikode study) by timing gait over a fixed distance. The utility of gait variables other than velocity in the context of cognitive decline are being elucidated in our and other studies, and we hope to provide more results of the cognitive-motor assessments and associations from the Kerala-Einstein study in the future. The similarity of our results in the Bronx and Kerala suggest that despite differing ethnic backgrounds, cultural settings, and lifestyle characteristics, the association between gait and cognition in the elderly is global and these simple clinical assessments have cross-cultural applicability. Cognitive-motor associations should be further explored to improve current diagnostic, risk assessment, and therapeutic approaches for patients presenting for evaluation of cognitive symptoms.
